# Whole-genome sequence, SNP chips and pedigree structure: building demographic profiles in domestic dog breeds to optimize genetic-trait mapping

**DOI:** 10.1242/dmm.027037

**Published:** 2016-12-01

**Authors:** Dayna L. Dreger, Maud Rimbault, Brian W. Davis, Adrienne Bhatnagar, Heidi G. Parker, Elaine A. Ostrander

**Affiliations:** 1 Cancer Genetics and Comparative Genomics Branch, National Human Genome Research Institute, National Institutes of Health, Bethesda, MD 20892, USA; 2 Institut de Génétique et Développement de Rennes, Rennes 35043, France; 3 PIC North America, Hendersonville, TN 37075, USA

**Keywords:** Population, Homozygosity, Canine, Inbreeding

## Abstract

In the decade following publication of the draft genome sequence of the domestic dog, extraordinary advances with application to several fields have been credited to the canine genetic system. Taking advantage of closed breeding populations and the subsequent selection for aesthetic and behavioral characteristics, researchers have leveraged the dog as an effective natural model for the study of complex traits, such as disease susceptibility, behavior and morphology, generating unique contributions to human health and biology. When designing genetic studies using purebred dogs, it is essential to consider the unique demography of each population, including estimation of effective population size and timing of population bottlenecks. The analytical design approach for genome-wide association studies (GWAS) and analysis of whole-genome sequence (WGS) experiments are inextricable from demographic data. We have performed a comprehensive study of genomic homozygosity, using high-depth WGS data for 90 individuals, and Illumina HD SNP data from 800 individuals representing 80 breeds. These data were coupled with extensive pedigree data analyses for 11 breeds that, together, allowed us to compute breed structure, demography, and molecular measures of genome diversity. Our comparative analyses characterize the extent, formation and implication of breed-specific diversity as it relates to population structure. These data demonstrate the relationship between breed-specific genome dynamics and population architecture, and provide important considerations influencing the technological and cohort design of association and other genomic studies.

## Abstract (precis)

**Summary:** Successful application of whole-genome sequencing and genome-wide association studies for identifying both loci and mutations in canines is influenced by breed structure and demography, motivating researchers to generate breed-specific strategies for canine genetic studies.

## INTRODUCTION

Early mapping studies utilized a combination of pedigree and linkage analyses to find genes important in disease susceptibility in dogs (i.e. Acland et al*.*, 1998; Cattanach et al*.*, 2015; Jónasdóttir et al*.*, 2000; Yuzbasiyan-Gurkan et al*.*, 1997). The construction of a canine genetic map, based on a large number of informative families, was key to the success of these experiments (Mellersh et al*.*, 1997; Neff et al*.*, 1999; Wong and Neff, 2009; Wong et al*.*, 2010). Early studies aimed at mapping disease traits similarly relied on the ability to collect samples from large informative pedigrees. Indeed, one of the unique advantages of domestic dogs for mapping traits has been the availability of large families, with single stud dogs often producing dozens of litters (Benson et al*.*, 2003; Jónasdóttir et al*.*, 2000). Such resources have been used to generate estimates of genetic heritability for many disorders and behaviors (i.e. Cooper et al*.*, 2014; Lappalainen et al*.*, 2015; Todhunter et al*.*, 2005; Persson et al*.*, 2015). Similarly, the effect of genetic bottlenecks and inbreeding on disease (i.e. Pedersen et al*.*, 2015; Reist-Marti et al*.*, 2012; Wilson et al*.*, 2013), together with overall trends in genetic diversity among purebred dogs (Hayward et al*.*, 2016; Lewis et al*.*, 2015), have all been investigated with single-nucleotide polymorphism (SNP) or microsatellite studies.

Genome-wide association studies (GWAS) carried out using SNP markers on chips allow for the identification of loci potentially associated with causation, using populations rather than large families. Although published reports describe loci identified from GWAS associated with morphologic traits (i.e. Cadieu et al*.*, 2009; Drögemüller et al*.*, 2008; Hayward et al*.*, 2016; Karlsson et al*.*, 2007; Parker et al*.*, 2009; Schoenebeck et al*.*, 2012; Vaysse et al*.*, 2011; Wolf et al*.*, 2014), disease susceptibility (reviewed in Lequarré et al*.*, 2011; Parker et al., 2010; Schoenebeck and Ostrander, 2014) and even behavior (Dodman et al*.*, 2010; Tiira et al*.*, 2012; Våge et al*.*, 2010), only a few such studies have been able to pinpoint precise mutations. The current standard for canine SNP assays – the Illumina HD chip, with 173,662 potential data points – is of limited utility due to low SNP density in many genomic regions, differential probe affinity, SNP ascertainment bias and the ability to generate only biallelic single-nucleotide variant (SNV) data. Long stretches of linkage disequilibrium (LD) characterize the dog genome, further reducing the utility of GWAS (Lindblad-Toh et al*.*, 2005; Sutter et al*.*, 2004). Finally, because each breed has a unique history, and therefore unique patterns of genomic diversity, GWAS studies are of varying success in correctly identifying loci in different breeds or unique lineages (Björnerfeldt et al*.*, 2008; Boyko et al*.*, 2010; Quignon et al*.*, 2007; vonHoldt et al., 2010).

Canine researchers are increasingly turning to whole-genome sequencing (WGS) to supplement the limitations associated with the less data-dense methods of pedigree analysis and SNP chip mapping. Success stories include studies of domestication (Axelsson et al*.*, 2013; Freedman et al*.*, 2014; Wang et al*.*, 2013), genome architecture (Auton et al*.*, 2013), trait selection and adaption (Gou et al*.*, 2014; Marsden et al*.*, 2016; Wayne and vonHoldt, 2012), and disease susceptibility (i.e. Decker et al*.*, 2015; Drögemüller et al*.*, 2008), among others. Variation within dog WGS data, combined with the unparalleled diversity of phenotypes in the dog, provides a unique lens for how genomic underpinnings influence organismal variation. WGS data, once obtained, can also be utilized beyond the initial study for which it was generated, including any hypothesis-driven analyses in which genomic signatures can inform biological questions.

Studies of how breed structure and history can be integrated with industry-standard use of SNP chip analysis and WGS to design the most successful canine genetic studies have yet to be explored. In this paper we consider all of the above in the context of many breeds of differing population substructure, demonstrating that, although a combination of approaches is optimal, population traits can dramatically impact how each should be applied. We define metrics through which population structure can be compared between breeds and determine how that structure should be interpreted with regard to study design and cohort assembly.

## RESULTS

### Genomic atlas for representation of dog breed diversity

Selection of breeds for pedigree, SNP and WGS analysis focused both on the availability of data as well as representation of diverse breeds as defined by breed type, history, geographical origin and modern popularity. To avoid selection bias, considerable effort was given to equal representation of breed type, function and size. The pedigree data reflect the current status of each breed in the United States (US), as well as the impact of historical changes such as global importation, breed registry recognition and trends in physical type or variety. There are seven historical American Kennel Club (AKC) breed groupings (toy, sporting, terrier, hound, herding, working and non-sporting) that categorize recognized breeds by their traditional function, type or purpose (Club, 2006). The pedigree analysis represents six of these groups, whereas the SNP and WGS analyses include breeds from all seven groups. The non-sporting group has the lowest representation overall, with seven breeds for both the SNP and WGS datasets, and no breeds in the pedigree data. The working group has the highest representation in the SNP and WGS datasets, with 19 and 16 breeds, respectively.

Combined, the WGS, SNP and pedigree datasets represent 112 dog breeds. Genetic variation at 1,510,327 LD-pruned loci aggregated from WGS of 90 dogs representing 80 breeds was analyzed, providing an impressive breadth of diversity by which to compare breeds and individuals. Complementing this, ten dogs from each of 80 breeds were genotyped using the current industry-standard canine HD SNP array with 173,662 potential sites of variation, providing definition within breeds, but potentially lacking in private variation. The 11 breeds utilized in the pedigree analysis were selected to reflect a range of breed population structures resulting from influences of time, geography and human intervention.

### Assessment of inbreeding coefficients by data type

The breed average inbreeding coefficient (*F*) was calculated three ways: (1) pedigree analysis from five generations, ten generations or the entire breed reference pedigree; (2) from SNP genotype homozygosity analysis averaged over multiple dogs per breed; and (3) from WGS homozygosity analysis of one dog per breed. Calculating *F* from five- and ten-generation pedigrees evaluates recent trends in inbreeding that occurred at some point after initial breed formation. Additionally, it accounts for differences in pedigree depth when compared to the complete reference population, because there was a large range in the number of effective generations (*g*_e_) for pedigree breeds. All pedigree breeds, however, did have *g*_e_ values greater than ten (Table 1).

**Table 1.**
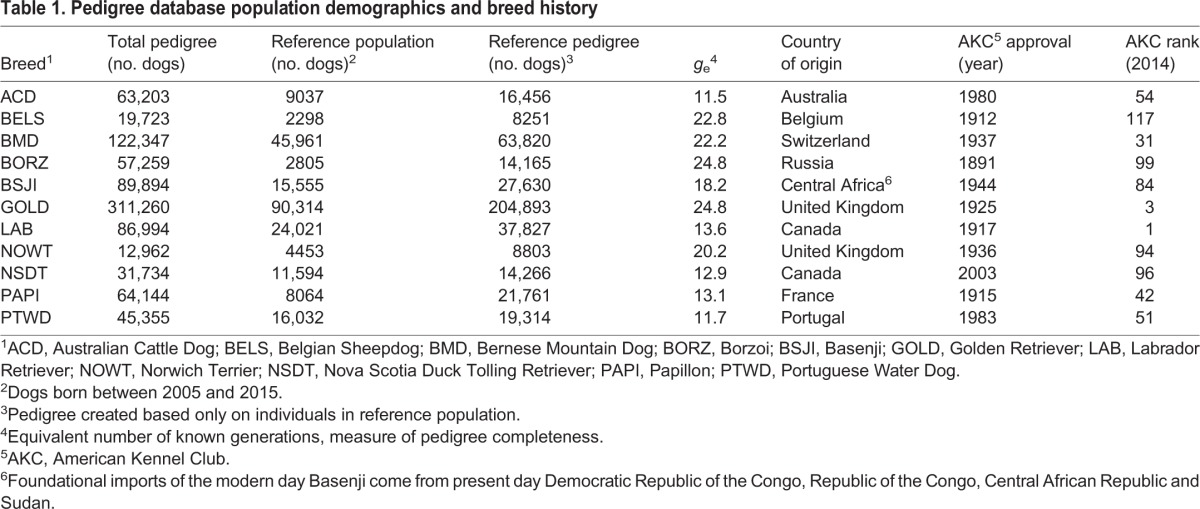
**Pedigree database population demographics and breed history**

Pedigree-based inbreeding coefficients ranged from 0.059 (Papillon) to 0.267 (Norwich Terrier) for whole-pedigree data, 0.051 (Papillon) to 0.251 (Nova Scotia Duck Tolling Retriever) when considering ten-generation pedigrees, and 0.022 (Bernese Mountain Dog) to 0.064 (Belgian Sheepdog) for five-generation pedigrees (Table 2). *F*-value calculations from both SNP genotypes and WGS data are higher than the pedigree analysis across all but the youngest breed (Nova Scotia Duck Tolling Retriever), with the SNP analysis showing a range of 0.179 (Papillon) to 0.536 (Basenji), and WGS *F*-values ranging from 0.118 (Portuguese Water Dog) to 0.571 (Basenji) (Table 2). Comparing *F*-values from subsets of the pedigrees, we observed the largest inbreeding coefficients when using the entire reference pedigree and the smallest inbreeding coefficients when examining only the most recent five generations in all breeds. Using only the most recent five generations reduces the across-breed range of *F*-values to only a span of 0.042 points compared to 0.208 and 0.200 points in the whole-pedigree and ten-generation calculations, respectively. This flattening of the values indicates that short-range pedigrees cannot account for the relationships between the earlier ancestors and therefore are no longer representative of the breed.

**Table 2.**
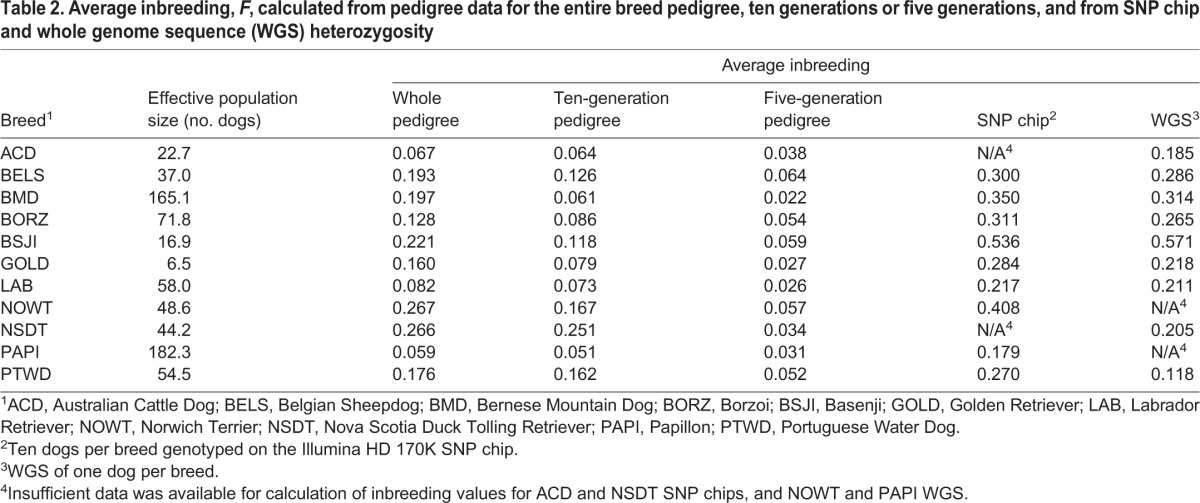
**Average inbreeding, *F*, calculated from pedigree data for the entire breed pedigree, ten generations or five generations, and from SNP chip and whole genome sequence (WGS) heterozygosity**

Comparing only the WGS and SNP data across 50 breeds, we observed a range of *F*-values calculated from the WGS of 0.488, from a minimum of 0.084 (Beagle) to a maximum of 0.571 (Basenji), and a range in SNP-based *F*-values of 0.423, from 0.113 (Chihuahua) to 0.536 (Basenji). The full list of *F*-values is shown in Table S1. Breed rankings of SNP- and WGS-calculated *F*-values showed positive significant correlation (*t*=6.179, *P*=1.24×10^−7^); however, neither the SNP nor the WGS *F*-values correlate with pedigree-based inbreeding coefficients (Table 3).

**Table 3.**
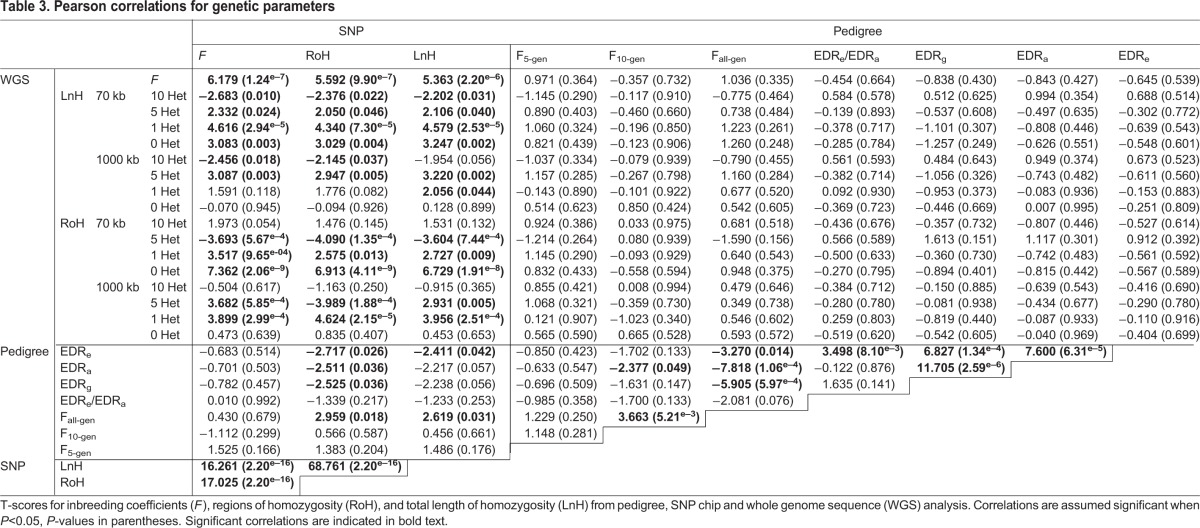
**Pearson correlations for genetic parameters**

Effective population size (*N*_e_), is the number of individuals in a population who contribute to offspring in the next generation, or the number of breeding individuals that would be required to explain the diversity apparent in a given generation. We hypothesized that this would vary strongly between breeds, because many breeds have undergone unique bottlenecks. In this case, *N*_e_ is measured as the change in the inbreeding value of a reference population with that of their parents' generation, and ranged from 6.5 (Golden Retriever) to 182.3 (Papillon) when measured from pedigree data. Using SNP data, the *N*_e_ was calculated for each breed over a time span of 13 to 995 generations prior to the acquisition date of the samples. The most recent *N*_e_ values, dated 13 generations ago, ranged from 53 (Bull Terrier) to 230 (Chihuahua). That is, at a reference point of 13 generations ago, the Bull Terrier had a reference population size of 53 dogs and the Chihuahua had an effective population size of 230 dogs.

**Fig. 1.**
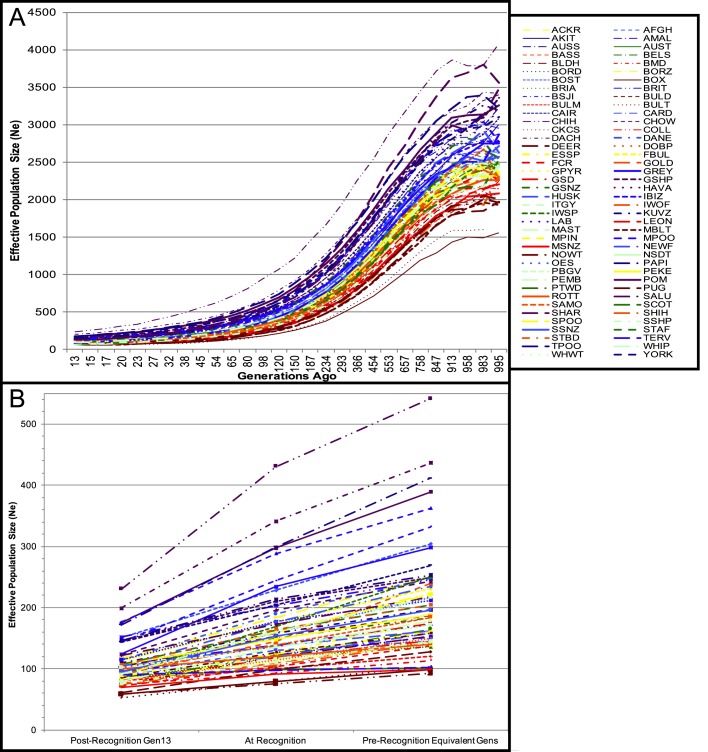
**Effective population size (*N*_e_) calculated from SNP genotypes.** (A) Change in *N*_e_ (number of dogs) per breed from 13 to 995 generations past. (B) Normalized values of *N*_e_ for each breed in the year that they were recognized by the American Kennel Club (AKC), the most recent calculated generation, and the time point prior to breed recognition equal to the span of time from breed recognition to present. The abbreviations used for each breed can be found in Table S1.

For each breed, *N*_e_ values decrease in an approximately exponential rate from distant past to present (Fig. 1A). Although the average slope of each breed-specific *N*_e_ curve ranges from 1.52 to 3.92, the breeds are each characterized by a unique *N*_e_ value at any given generation point. The data was normalized relative to the breed age, as determined by the AKC date of breed recognition, and a generation interval of 3.76 years (Windig and Oldenbroek, 2015) (Fig. 1B). The rate of change for *N*_e_ was calculated for time points from generation 13 to the year of registration, and from the year of registration to an earlier time point equivalent to the amount of time between generation 13 and the recognition year. The *N*_e_ at the time of AKC breed recognition ranged from 75 (Norwich Terrier) to 430 (Chihuahua). The difference between the slopes in *N*_e_ pre-AKC recognition and post-AKC recognition range from −1.77 (Basset Hound) to 5.16 (Chihuahua). The Basset Hound had a pre-AKC recognition slope indicating a loss of 4.21 breeding dogs per generation, whereas the post-AKC recognition slope showed a loss of 2.44 breeding dogs per generation. The Chihuahua had a pre-AKC recognition slope indicating a loss of 6.44 breeding dogs per generation, and a post-AKC recognition slope indicating a loss of 11.60 breeding dogs per generation. This can further be interpreted as the Basset Hound experiencing greater reduction in effective population size prior to breed recognition, and a lesser rate of reduction of breeding individuals after breed recognition. Conversely, the Chihuahua demonstrates the opposite scenario, with a larger generational decrease in effective population size after breed recognition, compared to before breed recognition. There is no significant correlation between the reference population pedigree *N*_e_ and the SNP-based generation-13 *N*_e_ (*P*=0.166), with the pedigree *N*_e_ values calculated as consistently lower than is revealed by SNP analysis.

### Population dynamics from purebred pedigree analysis

**Fig. 2.**
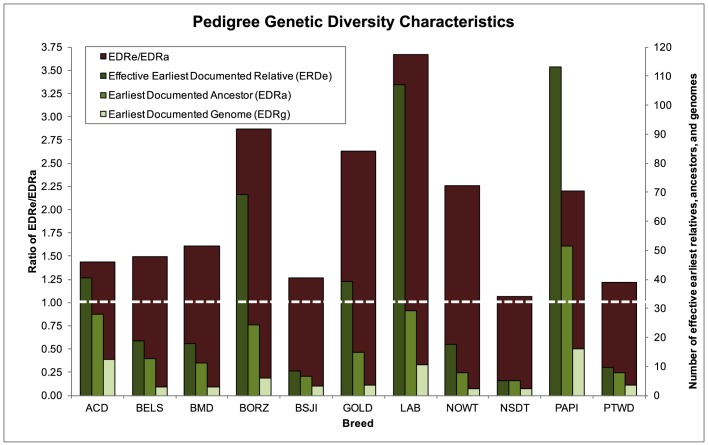
**Genetic diversity parameters reflected in the pedigree databases.** The ratio of effective to actual ancestors (*f*_e_/*f*_a_) is measured on the primary axis and suggests the occurrence of a bottleneck event when over one, which is indicated with a dashed white line. The abbreviations used for each breed can be found in Table S1.

The earliest documented relatives that contributed genetically to the reference population for each breed (EDR_e_) was used as a means to estimate the original diversity of the breed at the earliest recorded point in breed history. Calculated from the most recent generation, EDR_e_ ranged from 5.2 (Nova Scotia Duck Tolling Retriever) to 113.1 (Papillon) (Fig. 2). When expanding the potential influencing relatives to include any ancestors, dependent on their marginal contribution to the reference population, the number of effective ancestors (EDR_a_) was shown to range from 4.9 (Nova Scotia Duck Tolling Retriever) to 51.4 (Papillon). According to the metrics of EDR_e_ and EDR_a_, and the number of effective genomes (EDR_g_) ranging from 2.2 (Norwich Terrier and Nova Scotia Duck Tolling Retriever) to 16.1 (Papillon), the Nova Scotia Duck Tolling Retriever displays the lowest amount of genetic diversity of all the pedigree breeds, whereas the Papillon shows the highest. The ranked order of the least to most diverse breeds remains nearly consistent whether considering using either the EDR_e_ or EDR_a_ metric, except with regard to the Australian Cattle Dog and Borzoi, which switch order in the rankings when considering non-founder ancestor contribution (EDR_a_). This suggests that, although the Australian Cattle Dog is less genetically diverse than the Borzoi when considering EDR_e_, there is notable influence of non-founder ancestors in the Borzoi breed that result in their EDR_a_ value dropping below that of the Australian Cattle Dog. A ratio of EDR_e_/EDR_a_ of greater than one is indicative of a bottleneck event in the history of the breed. Although minimal in the Nova Scotia Duck Tolling Retriever (1.06), all breeds analyzed showed some indication of a bottleneck event, with the strongest event occurring in the Labrador Retriever (3.67).

### Genomic analysis of homozygosity

**Fig. 3.**
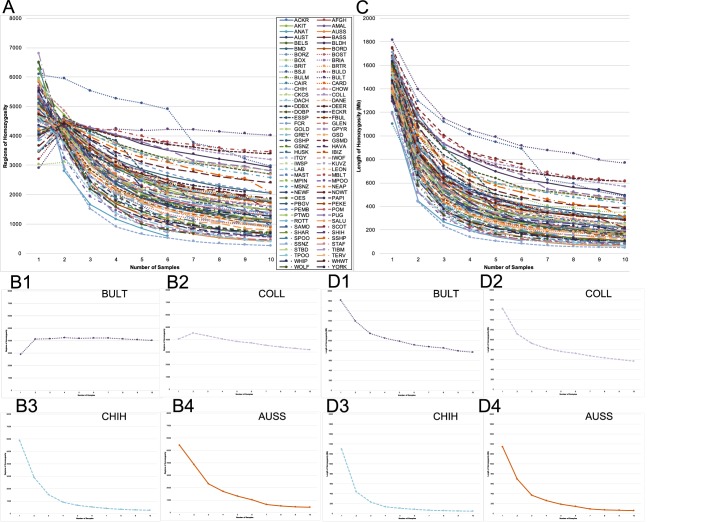
**Shared regions of homozygosity (RoH) and length of homozygosity (LnH) from SNP chip genotypes, re-calculated with sequential addition of single same-breed dogs.** (A,B) RoH; (C,D) LnH. The labels and values of the *x*- and *y*-axes in B and D are the same as in A and C, respectively. A and C represent each of 80 breeds, and display the overall pattern of loss of private homozygosity with the inclusion of additional dogs. B and D are homozygosity decay curves for breeds at the extreme values of high shared homozygosity and low rate of decay [Bull Terrier (BULT) and Collie (COLL)] and low shared homozygosity and high rate of decay [Chihuahua (CHIH) and Australian Shepherd (AUSS)]. Individual breed graphs for LnH decay are available in Fig. S2. The abbreviations used for each breed can be found in Table S1.

To estimate the level of breed homozygosity, it was necessary to filter out the regions of private homozygosity, i.e. those regions that are homozygous in an individual dog but might be heterozygous in other dogs of the same breed, and thus not an indicator of breed-specific homozygosity. For the purpose of this study, homozygous regions present in all sampled individuals of a given breed are denoted ‘shared’. Shared regions of homozygosity (RoH) and length of homozygosity (LnH) are therefore common across all representatives of a breed. These were calculated incrementally for each SNP-genotyped breed by randomly adding individuals and recalculating shared homozygosity until all members of the breed, to a maximum of ten, were included (Fig. 3; Fig. S2). In 23 of the 80 SNP-genotyped breeds, shared RoH temporarily increased with the addition of the second dog but shared LnH decreased, indicating that large private runs of homozygosity, present in the initial dog, were broken into smaller pieces by the addition of a second dog. At three through ten dogs, both the RoH and the LnH values decreased by exponentially lesser extents with each new additional dog, such that the tenth dog reduced the first dog's private LnH by between 0.28% (Miniature Poodle) and 7.18% (Shetland Sheepdog) (Fig. 3).

Although the same general pattern was observed in each breed, the rate at which each breed decreased in terms of shared LnH varies. The rate of decay for shared LnH, i.e. the proportion by which shared LnH decreased from the first-dog private LnH with each additional dog, ranged from 0.1996 (English Springer Spaniel) to 0.6065 (Miniature Poodle), with a mean of 0.4098 and s.d. of 0.085 (Table 4).

**Table 4.**
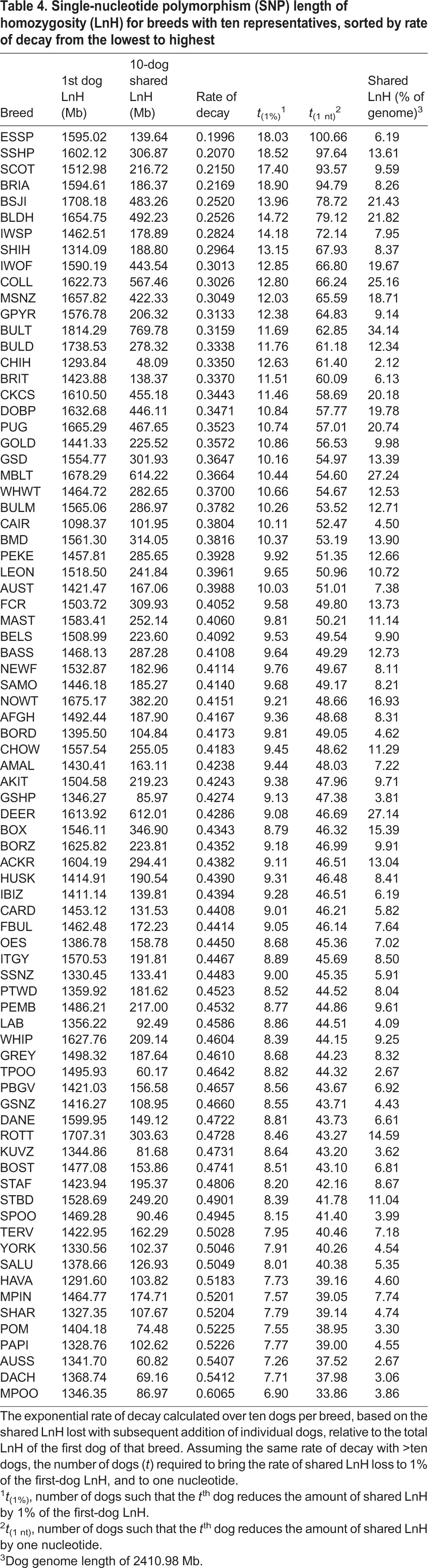
**Single-nucleotide polymorphism (SNP) length of homozygosity (LnH) for breeds with ten representatives, sorted by rate of decay from the lowest to highest**

Because WGS is often available for only a single individual of a given dog breed due to cost considerations, we compared the relative value and utility of SNP chip genotyping on multiple dogs versus WGS analysis of a single dog. The WGS data was first pruned to remove the SNPs in LD with one another. Because the average spacing of SNPs on the Illumina Canine HD SNP chip is approximately 14 kb and the WGS variants are, on average, only 306 bp apart, homozygosity in the WGS was calculated based on length of region rather than number of SNPs. Additionally, SNP chip analysis might call a region as homozygous despite the potential for heterozygosity between genotyped SNPs, whereas, in WGS, essentially every SNP is genotyped, leaving no missed heterozygosity. In order to compare these two disparate datasets, multiple parameters (see Materials and Methods) were used to calculate homozygosity from the WGS data of single-dog breeds varying both the window size and the allowed heterozygosity within the window. Using the metrics of a 70 kb window and zero heterozygotes, approximately equivalent to a five SNP window from the chip data calculations, the single-dog WGS predicted greater LnH than the shared SNP chip LnH values across all breeds. The shared values of LnH from the SNP chip analysis most closely resemble the single-dog WGS LnH values when calculated with parameters of 1000 kb minimal length and zero allowed heterozygotes, although the breed pattern is not correlated (*P*=0.899).

**Fig. 4.**
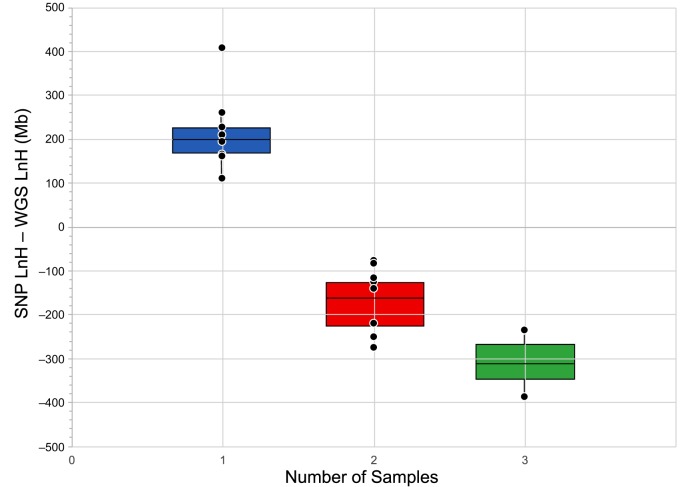
**Difference between single-nucleotide polymorphism (SNP) and whole-genome sequence (WGS) shared length of homozygosity (LnH).** Based on one dog, LnH is greater for SNP data than for WGS data. For two or three dogs, however, shared LnH from SNP data is less than for the same breeds from WGS data.

In addition to single breed representatives, the WGS collection included six breeds for which two dogs were sequenced and two breeds for which three dogs were sequenced. For these breeds, shared LnH and RoH were calculated in the same manner used to assess shared values in the SNP-genotyped breeds. Across all eight breeds, the LnH of the first dog was lower using WGS data than for the SNP analyses. However, the relationship was reversed with the addition of the second dog from each breed such that the shared LnH between two dogs was greater using WGS data than SNP genotypes. Fig. 4 demonstrates the difference between shared LnH of SNP and WGS data between one, two and three dogs. The single-dog LnH is, on average, 216 Mb longer based on SNP data than WGS data. When calculated using data from two dogs per breed, however, the shared LnH is, on average, 162 Mb longer using WGS data than SNP data. In addition, utilization of WGS for calculation of shared RoH removed an artifact observed in the SNP data, where the shared RoH increased transiently when two dogs were considered due to the artificial reduction of RoH with data generated by only one dog. Although shared RoH measured by SNPs increased with the second dog for Bernese Mountain Dog and Rottweiler breeds, the shared RoH decreased with the addition of a second dog in all breeds based on WGS.

To determine whether pedigree analysis can predict genomic homozygosity measures of population diversity, calculated from family pedigree history (*F*), SNP chip genotypes (*F*, *N*_e_, RoH and LnH) and WGS (*F*, RoH and LnH) were compared using Pearson's correlation (Table 3). To obtain a point of comparison to validate the use of SNP data across multiple individuals of the same breed versus single-dog SNP data, correlation calculations were performed based on WGS measures of homozygosity, both with randomly selected single breed representative SNP data and shared SNP data across multiple breed representatives. By allowing WGS homozygosity parameters to vary (70 kb or 1000 kb minimum length; 0, 1, 5 or 10 heterozygotes), and randomly selecting one dog per breed for the SNP homozygosity calculations, there was a significant positive correlation (data not shown) across the 51 common breeds based on single-dog SNP LnH and WGS LnH for short-range, low-heterozygote parameters (*P*_70 kb/5 het_=0.0166, *P*_70 kb/1 het_=3.22×10^−6^, *P*_70 kb/0 het_=1.20×10^−4^), versus SNP LnH and WGS LnH for long-range, moderate-heterozygote parameters (*P*_1000 kb/5 het_=5.10×10^−4^, *P*_1000 kb/1 het_=9.53×10^−3^).

A significant positive correlation was observed between SNP- and WGS-based inbreeding coefficients (*P*=1.24×10^−7^) for the 51 breeds common to both data sets; however, none of the pedigree-based inbreeding coefficients correlated with the equivalent values for SNP chip or WGS, for which there were ten and nine common breeds, respectively. Both shared SNP-based measures of homozygosity were positively correlated with whole-pedigree inbreeding coefficients (*P*_RoH_=0.018, *P*_LnH_=0.031) and WGS homozygosity values when parameters dictated small minimal lengths (70 kb) of homozygosity, and allowed for zero to five heterozygotes. The SNP-based calculation of *N*_e_ showed significant negative correlation with SNP RoH (*P*=3.41×10^−11^), LnH (*P*=9.22×10^−10^) and *F* (*P*=4.51×10^−8^). However, no significant correlation was observed between the SNP-based *N*_e_ and pedigree-based *N*_e_ values (*P*=0.166). The pedigree analyses did not correlate with any of the WGS RoH or LnH calculations. Shared LnH calculated from SNP chips correlates most closely with the LnH from WGS analyses using a 70 kb window, allowing for a heterozygote at only one locus. Shared RoH calculated from SNP chips correlates most closely with WGS using a 70 kb window and no heterozygotes (Table 3). Although the same patterns of correlation were seen for LnH and RoH when considering WGS data and either single-dog SNP data or multi-dog shared SNP data, the correlations were of highest significance between WGS and single-dog SNP values. However, considering the observed variation between individuals, the shared SNP homozygosity values better represent the homozygosity status of an entire breed.

## DISCUSSION

Although the diversity of the dog is increasingly prized for its contribution to human health and mammalian biology, we, like others, observe that the source of this diversity, namely breed structure, presents barriers and complications (Lindblad-Toh et al*.*, 2005; Marsden et al*.*, 2016; Schlamp et al*.*, 2016; Wijnrocx et al*.*, 2016). Modern domestic dog breeds exist because humans have carefully selected traits of desired purpose. Importantly, they have been, and continue to be, influenced by geographic, cultural and societal forces (Björnerfeldt et al*.*, 2008; Quignon et al*.*, 2007). The work presented here examines variables of inbreeding and homozygosity in a large and comprehensive set of dog breeds through parallel use of pedigree data, genome-wide SNP genotyping and WGS. Specifically, we compare data from extended pedigree analysis, genotyping with an SNP chip of 173,622 potential data points, and WGS with an average depth of 27.79X.

We found that each dog breed has a unique profile of genome diversity, varying by amount of total homozygosity as well as number and size of homozygous regions. Likewise, although we observe variation between members of the same breed, multiple individuals from a single breed can be combined to obtain an accurate reflection of breed-specific homozygosity and knowledge regarding fluidity of variation within breed confines. This allows us to define metrics that inform the design of canine genetic studies while also allowing us to develop an understanding of the intricate complexity of the diversity of dog breeds. Individual diversity metrics are provided for over 100 breeds as a resource for investigators in the field (Table 4; Tables S1, S2).

### Population characteristics reflect breed history

**Fig. 5.**
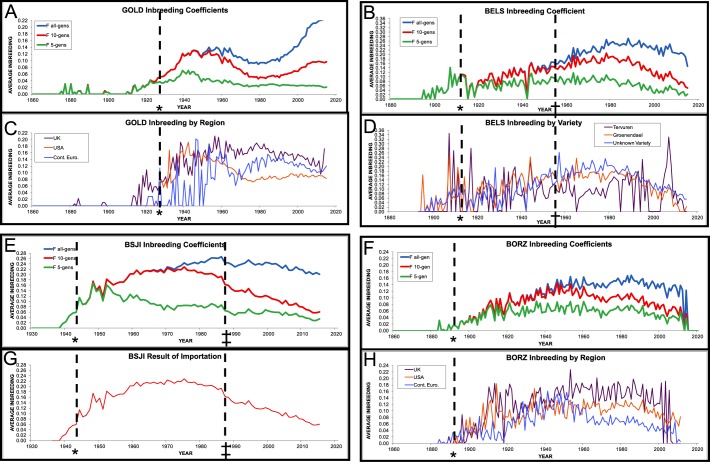
**Graphical representation of inbreeding coefficients.** (A,C) Golden Retriever (GOLD); (B,D) Belgian Sheepdog (BELS); (E,G) Basenji (BSJI); (F,H) Borzoi (BORZ). Inbreeding coefficients are calculated over the entire reference pedigree, and including only ten generations or five generations (A,B,E,F). Populations were split by geographic region (C,H) or by breed variety (D) to demonstrate influences on inbreeding values. *Year of breed recognition by the American Kennel Club (AKC). ^†^Year at which the AKC classified the Belgian Tervuren and Belgian Sheepdog as separate breeds. ^‡^Year at which the Basenji studbook allowed for limited new imports of dogs from Africa to the USA.

As expected, pedigree records for all breeds were erratic and often incomplete prior to breed establishment in their country of origin. In spite of these inconsistencies, considerable across-breed patterns, as well as breed- or situation-specific fluctuations, were identified through pedigree analysis (Fig. 5). Breed recognition by a kennel club registry both requires and facilitates pedigree tracking, thus improving records for each breed relative to the point at which the origin registry granted breed status. Concurrent with establishment of a breed, we observe that levels of inbreeding increase steadily and often immediately. Additional increases are noted when the breed achieves registry recognition. This implies that organization of a breed reduces the available gene pool, first by closing the registration database or ‘studbook’ to the introduction of non-breed-associated genetic variation, imposing an artificial population bottleneck. In addition, it provides a merit system through organized competitions in the sense that a small number of individuals ‘win’ at dog shows and the genetic contribution of those popular dogs are overrepresented in subsequent generations of the breed, recognized as the ‘popular sire effect’, thus decreasing variability in the breed pool still further. This is displayed clearly in the ten-generation and all-generation inbreeding graphs of the Australian Cattle Dog, Golden Retriever, Norwich Terrier, Bernese Mountain Dog, Borzoi and Basenji (Fig. S1). This trend is likewise reflected in pedigree-calculated *N*_e_ values, where the Golden Retriever, despite being ranked among the top five most popular dog breeds by the AKC from 1993 to 2015 (www.akc.org), has an *N*_e_ of 6.5, and the Papillion, with popularity ranking of 48th for 2015, records a substantially higher *N*_e_ of 182.3. The large range of *N*_e_ values does not necessarily coincide with the total number of breed individuals registered per year or the breed's relative popularity. Rather, it speaks to the general within-breed diversity of the breeds and the contribution of popular dams and sires to the subsequent generation.

In some cases, we observed that formation of the original breed club and creation of an official standard seemed to briefly increase the breed pool, thus transiently decreasing inbreeding values, perhaps by legitimizing and unifying previously distinct lines. This diversity is misleading, however, because dogs that seem unrelated at the five- to ten-generation level ultimately trace back to the same small number of founders. This is supported by the correlation between full-pedigree *F*-values and whole-genome homozygosity measures (*P*_SNP-LnH_=0.031; *P*_SNP-RoH_=0.018), concurrent with a lack of correlation with shorter-generation *F*-values (Table 3). The breed ranking of five-generation *F*-values compared to the larger pedigree analysis or to molecular measures of homozygosity suggests that examination of short-range pedigrees for information about the relationships between individuals in a breed will likely provide misleading information as to the status of the breed as a whole. With the exception of the youngest breed included (the Nova Scotia Duck Tolling Retriever, which seems to have reached a plateau), all analyzed pedigrees showed a peak in inbreeding post-AKC recognition, although the time required to reach that peak ranges from 9 (Labrador Retriever) to 69 (Papillon) years.

When ranking breeds by level of inbreeding and ancestor contributions, the Nova Scotia Duck Tolling Retriever shows the lowest diversity in a majority of categories (ten-generation pedigree inbreeding, EDR_e_, EDR_a_, EDR_g_), whereas the Papillon demonstrates the highest level of diversity, as calculated from the same metrics, plus whole-pedigree inbreeding measures. These values reflect the historical account of breed formation for these breeds. Whereas the Papillon has a diffuse record of origin, spanning much of Western Europe and staking claim to the small spaniel-like dogs represented in artistic renderings from the 16th century (https://www.papillonclub.org/History/Welcome.html), the Nova Scotia Duck Tolling Retriever is the result of a concerted breeding effort to produce dogs that display a very specific game-luring behavior to aid hunters, centered in the Maritimes of Canada during the 19th century (http://nsdtrc-usa.org/breed/history/).

An EDR_e_/EDR_a_ ratio greater than one indicates the presence of a bottleneck event in the history of a population. Although all breeds achieved a ratio score greater than one, it was most prominent (greater than two) in Borzoi, Golden Retriever, Labrador Retriever and Norwich Terrier (Fig. 2). By evaluating breed history and years of birth of influential ancestors, we can speculate as to the cause of most bottlenecks. We find that, in each case, 10-29% of the most influential ancestors in a breed could be traced to a 5- to 10-year period. These time periods coincide with import/export events in the case of the Borzoi, the recognition of the breed or breed club for the Golden Retriever and Norwich Terrier, and a drastic increase in popularity and population size in the Labrador Retriever.

The databases used for this study were largely US-centric, hence bottleneck events at breed formation or coincident with importation of breeds to the US would be expected, but were not necessarily observed for all breeds. This likely represents events that predate the earliest available records. For example, the first Basenjis were imported to the US around 1941, and our data show that a single male, born in 1939, accounts for 30.3% of the genetic diversity observed in the current reference population. The importation of dogs from central Africa to the US would almost certainly have resulted in a bottleneck event, but this cannot be confirmed without accurate pedigree data for the African Basenji. We can, however, recognize a distinctive drop in inbreeding values in the Basenji during the late 1980s, corresponding with the time at which new African imports of Basenjis were allowed for registration with the AKC. The intensity of the bottleneck does not correlate with any of the molecular measures of population structure. Independently, however, the number of RoH measured by SNP chip showed significant negative correlation with all measures of earliest documented relatives: EDR_e_ (*t*=−2.717, *P*=0.026), EDR_a_ (*t*=−2.511, *P*=0.036) and EDR_g_ (*t*=−2.525, *P*=0.036). Therefore, a decreased number of effective earliest documented relatives, ancestors and genomes correlated with an increase in shared RoH as measured by SNP chip.

### Implications of homozygosity decay

When shared homozygosity measures are calculated within a breed by sequentially increasing the number of dogs in the dataset from one to ten individuals, the LnH that is lost with each additional dog is assumed to be private to the individual or not fixed within that breed. Conversely, there is a point at which the homozygosity identified as shared across a pool of individuals is likely shared within the entire breed (Fig. 3). By utilizing the extent to which the LnH decreases with each additional dog added to the shared LnH calculation, an exponential rate of decay can be calculated, which can in turn be used to predict the number of individuals required to represent the entire range of variation within a given breed as inferred from the current SNP array. When these calculations were performed on 80 breeds using data from SNP genotyping, the rate of decay ranged from 0.1996 (English Springer Spaniel) to 0.6056 (Miniature Poodle). Thus, as each dog is sequentially added to the data set, the shared LnH decreases by 19.96% in the English Springer Spaniel or 60.56% in the Miniature Poodle (Table 4).

With a normal distribution across 80 breeds, and a s.d. of 0.085, 54 breeds have a rate of decay within 1 s.d. of the all-breed mean. The breeds located at the extremes of 3 s.d. from the mean include the Miniature Poodle (0.6056) at the high end and the English Springer Spaniel (0.1996), Shetland Sheepdog (0.2070), Scottish Terrier (0.2150) and Briard (0.2169) at the low end (Table 4). When designing studies, such as GWAS, that utilize SNP data, these decay rates can be implemented to predict the number of dogs in a breed that would be required to represent the full range of variation within that breed. The breeds in the extreme left tail of the decay rate distribution (English Springer Spaniel, Shetland Sheepdog, Scottish Terrier, Briard) would require 17 to 19 individuals to bring the per-dog loss of LnH down to 1% of the initial-dog LnH, or 93 to 100 individuals to bring the per-dog loss of LnH down to only one nucleotide. Conversely, the Miniature Poodle on the extreme right tail of the decay rate distribution requires only seven individuals to reach 1% of initial-dog homozygosity or 34 individuals to reach one nucleotide of homozygosity reduction (Table 4).

Caveats to the above, however, include the fact that decay rate does not speak to the overall amount of homozygosity contained within each breed, only the amount of variation between individuals within that breed. For example, the breed with the largest shared LnH is the Bull Terrier (769.78 Mb), whereas the breed with the smallest shared LnH is the Chihuahua (48.09 Mb) (Fig. 3). Comparatively, the decay rate difference between the Chihuahua (0.3350) and the Bull Terrier (0.3159) is only 1.91%, dictating that both breeds require approximately 12 dogs to represent 99% of the genomic variation within the breed, despite the difference in shared homozygosity of 721.69 Mb. This concept is important and, as a result, we provide breed-specific measurements of homozygosity as well as unique signatures of decay representing variation within 80 breeds (Table 4) as a utility to researchers in the field. Both of these variables are necessary to guide genome-based experimental design in pure-breed dogs because our data suggest that the degree to which individual dogs of the same breed vary in homozygosity in relationship to each other, reflected by the decay rate, is of at least equal importance to the overall shared homozygosity of a breed for determining required sampling size for mapping studies.

In order to test the capabilities of breed-specific homozygosity decay to predict appropriate cohort size, a small-scale proof-of-concept experiment was conducted. Genetic variation of the *RSPO2* gene has previously been associated with the wire or furnished coat type, a phenotype that is fixed and easily recognizable in many dog breeds (Cadieu et al*.*, 2009). The Miniature Poodle and Scottish Terrier are breeds that are near fixation for the furnishing *RSPO2* variant, and have high (0.6065) and low (0.2150) homozygosity decay rates, respectively. Likewise, the Papillon (high decay rate=0.5226) and Shetland Sheepdog (low decay rate=0.2070) are wild-type at the furnishings locus. The high decay rates suggest that between seven and eight dogs would be necessary to reduce the shared LnH to 1% of the initial, whereas the low decay rates indicate 17 to 19 dogs would be required to produce the same results. Using the ten dogs of each breed genotyped on the Illumina HD SNP array, a standard non-adjusted association analysis was conducted with PLINK software (Purcell et al*.*, 2007) using high-decay-rate Papillons as controls and high-decay-rate Miniature Poodles as cases, and separately, with low-decay-rate Shetland Sheepdog controls and low-decay-rate Scottish Terrier cases (data not shown). With ten cases and ten controls, the comparison of Papillon with Miniature Poodle assigned highest significance to the region surrounding *RSPO2* on CFA13. The equivalent analysis of low-decay-rate Shetland Sheepdog and Scottish Terrier breeds did not indicate significant association with *RSPO2*. Subsequently, with sequential reduction of one random dog per breed from the high-decay-rate analysis, the *RSPO2* region maintained the highest relative significance from *n*=10 through *n*=7, although the *P*-value did drop below genome-wide significance. Therefore, even under loose constraints, there is evidence for homozygosity decay rates to serve as predictive for trait-mapping ability.

### Observation of pedigree structure by molecular means

Population traits calculated through pedigree analysis did not correlate with genomic measures unless the entire reference pedigree was used. Pedigree-based inbreeding coefficients calculated over the entire reference pedigrees correlated significantly with the RoH (*t*=2.959, *P*=0.018) and LnH (*t*=2.619, *P*=0.031) calculated using SNP genotypes shared across ten dogs of each breed, but did not correlate at all with homozygosity measures of *F*, LnH or RoH derived from individual WGS. This would suggest that deep reference pedigree structure can be captured by SNP analysis of multiple breed individuals, but is not apparent in WGS analysis of only one individual. Likewise, five- or ten-generation pedigree analysis is not sufficient to elucidate the larger breed-specific population structure apparent in the entire pedigree reference populations.

### Comparison of WGS and SNP measures of homozygosity

In this study we compared the genome-wide estimates of breed-expected homozygosity based on SNP genotyping of several individuals versus WGS of one individual, two similarly priced methods currently in use among canine geneticists. Genome-wide and breed-specific estimates of homozygosity dynamics can be useful for designing association studies by aiding in determination of the number of individuals that will be necessary to identify the locus of interest.

When comparing breeds in which shared homozygosity was calculated from SNP data, a significant positive correlation was observed between *F*-values and shared LnH (*t*=16.261, *P*=2.20×10^−16^) and shared RoH (*t*=17.025, *P*=2.20×10^−16^) (Table 3), supporting the expectation that higher inbreeding coefficients result from increased homozygosity. Within that SNP-based data, increasing *F* in breeds with similar shared LnH (<1000 kb difference) increased RoH in 53.8% of breed pairs. However, increasing shared LnH in breeds with similar *F*-values (<0.1% difference) resulted in an increased RoH in 84% of the breed pairs. This suggests that increased inbreeding produces a greater number of homozygous regions, rather than increased length of pre-existing homozygous regions. This finding corroborates the previously discussed negative correlation between lower pedigree-based values of EDR_e_, EDR_a_ and EDR_g_ and increased SNP-based RoH.

Although correlation was observed between shared SNP LnH and single-dog WGS LnH over short regions, the significance of that measure varied greatly depending on the number of allowable heterozygotes or minimum length of the WGS regions assessed. For instance, the correlation value for SNP LnH with WGS LnH with a minimum length of 70 kb and one allowed heterozygote was *t*=4.579, *P*=2.53×10^−5^, whereas the correlation value for SNP LnH with WGS LnH of the same minimum length but with five allowed heterozygotes was *t*=2.106, *P*=0.040 (Table 3). Recognizing that calculating shared homozygosity over multiple individuals will reduce the effects of private homozygosity in SNP-based data, we applied the same process to small numbers of dogs for which WGS was obtained. As such, we could directly compare the difference in single-dog WGS homozygosity and two- or three-dog WGS shared homozygosity, and determine whether the patterns were reflective of those seen in comparable SNP-based calculations.

SNP-based homozygosity calculations rely on the expectation of long regions of LD to assume homozygosity between markers. This can lead to artificially reduced numbers and increased size of RoH if chip SNPs are homozygous while intervening regions contain variation, or are not reflective of heterozygosity in certain breeds, creating artificially inflated measures of LnH. Although WGS LnH for one dog per breed yielded lower values than observed from SNP-calculated shared LnH, the inclusion of two WGS dogs per breed resulted in shared LnH values greater than observed in the respective SNP genotyped breeds (Fig. 4). Because WGS has the potential to provide a more accurate representation of genomic metrics, these results indicate that the ∼170K SNP chip overestimates individual LnH and underestimates shared LnH, relative to WGS values. Owing to cost, WGS is usually applied to fewer individuals than SNP chip technology; however, the inclusion of two WGS dogs can reduce the LnH from one dog by 15.5% to 25.5%. In the two breeds for which we used three WGS dogs to calculate shared LnH, the third dog reduced the initial LnH by an additional 8% in each case. These values might, therefore, support a cost/benefit argument for the inclusion of WGS from two dogs per breed, instead of only one, when considering study design.

Despite the two genomic methods occasionally producing discordant values for breed-specific RoH and LnH, the significant correlation between the two distributions across all 50 breeds for which both SNP and WGS data was analyzed suggests that either SNP chip analysis or WGS of a small number of dogs would be sufficient to estimate degree of homozygosity when considered relative to other breeds. Although WGS from multiple individuals will produce continually more accurate data, through comparison to the database of breed homozygosity measurements provided here, single-dog sequencing homozygosity measurements can be sufficient to predict breed-wide genomic structure.

For the majority of dog breeds for which WGS data is publicly available thus far, generally one individual per breed has been sequenced (http://www.ncbi.nlm.nih.gov/sra). If multiple dogs have undergone WGS, it often reflects different geographic origins of the lineage and the utility of that data for designing a genomic study in one geographic region might be limited (Quignon et al*.*, 2007). The data employed in this study are restricted in large degree to purebred dogs sampled within the US. However, similar studies undertaken in European populations are likely to produce equally informative results, both for understanding breed genomic architecture among European breeds and for the design of association studies.

In this study, we leveraged data from three sources to study genome structure in dog breeds and, in doing so, have characterized the unique and extensive breed-specific population structure present in modern dog breeds so as to predict optimal study designs for genetic experiments. By combining comprehensive genotype and relationship data from these three separate technologies, we have produced a series of metrics that are directly applicable to studies of domestic dog breed structure and suggest how those metrics should be applied to the design of canine genetic studies. In summary, we propose that each study design be considered independently in terms of not only the expected inheritance pattern displayed, but also the breed in which the trait occurs. Cohort sizes for SNP analysis should reflect the breed-specific decay rates and levels of homozygosity displayed in Table 4. When considering WGS analysis, greater benefit is achieved by obtaining data from two individuals than is gained with the inclusion of a third individual of the same breed. The relative degree of inbreeding, effective population size and homozygosity from SNPs correlates with the inbreeding and short-range (70 kb) homozygosity rankings from WGS. However, pedigree-based metrics do not necessarily provide an accurate representation of the genetic population measurements. Although mode of inheritance and effects of penetrance, epistasis and pleiotropy all play a role in the outcome of an association study, we show that accurate assessment of breed-specific genomic topography is exquisitely valuable for determining the most effective sample size. We believe that these efforts to capture the distinctive and dynamic homozygosity landscape of over 100 pure dog breeds will serve as a platform upon which future research initiatives can be modeled.

## MATERIALS AND METHODS

### Pedigree datasets

Data used for pedigree analysis in this study were provided by private breed databases for the following 11 breeds: Australian Cattle Dog, Belgian Sheepdog, Bernese Mountain Dog, Borzoi, Basenji, Golden Retriever, Labrador Retriever, Norwich Terrier, Nova Scotia Duck Tolling Retriever, Papillon and Portuguese Water Dog. Due to breed history, the Belgian Sheepdog database also includes several Belgian Tervurens and Belgian Malinois, and the Golden Retriever database includes early contributing breeds including several Flat-coated Retrievers, Irish Setters and Labrador Retrievers. The abbreviations used for each breed throughout this study can be found in Table S1. Breeds for pedigree analysis were selected based on the availability of high-quality database information. To be entered into this study, the breed database had to include at least 10,000 individuals over >10 effective generations (*g*_e_), calculated as described in the Pedigree analysis section, and reflect the modern breeding population of the breed by including at least 10% of the AKC-registered dogs for that breed, determined by registration data for 2014. Exceptions were made for the Labrador Retriever and Golden Retriever breeds, which, due to their immense popularity, reflected only 0.47% and 6.55% of AKC registrations for those breeds, respectively. Despite this, the database reference population sizes for Golden Retriever and Labrador Retriever ranked first and third highest of included pedigree breeds.

Breed databases were predominantly compiled by volunteer enthusiasts using registries, studbooks, historical records, dog-show entries and personal breeder records. Initial pedigree analysis required an internal error check whereby improbable relationships are flagged for manual correction. Breeds were removed from study inclusion when the pedigree database contained insurmountable inconsistencies. When data regarding country or year of birth was missing from the raw database, estimated values were extrapolated based on equivalent data from siblings, offspring, dam and sire, and assuming a standard generation interval of 2 years. Demographics from the datasets are provided in Table 1, demonstrating that these breeds reflect the variation present in American populations of modern dog breeds with regard to country of origin, population size, popularity and history. Pedigree databases ranged in size from 12,962 (Norwich Terrier) to 311,260 (Golden Retriever) dogs.

The AKC began accepting breeds for registration in the 1880s and the earliest recognized pedigree breed in this study is the Borzoi from 1891 (http://www.akc.org/press-center/facts-stats/page-3/). The databases used in these analyses, however, record parentage as far back as the 1830s (Labrador Retriever), 1840s (Golden Retriever) and 1860s (Borzoi). Of the breeds included in the study, the Nova Scotia Duck Tolling Retriever was the most recent to gain recognition by the AKC, obtaining breed status in 2003. Pedigree data for this breed, however, dates back to 1930. North America is listed as the predominant region of birth or initial registration for all 11 breeds, representing between 37.04% (Norwich Terrier) and 76.29% (Portuguese Water Dog) of dogs within the pedigrees of the modern dogs. The dogs not listed as being born in North America derive primarily from Europe, Asia and Oceania.

All dogs born between 2005 and 2015 make up the reference population for each breed. The direct ancestors of those dogs were traced back through successive parent-offspring relationships to the earliest dogs within the respective database. Importantly, use of a reference population to represent the modern breed removes any individuals present within the database that do not genetically contribute to the development of the breed as it currently exists. Reference pedigrees, comprising the reference populations and their ancestors, ranged in size from 8251 (Belgian Sheepdog) to 204,893 (Golden Retriever) and covered between 11.5 (Australian Cattle Dog) and 24.8 (Golden Retriever and Borzoi) effective generations. All subsequent calculations were based on the reference pedigree.

### Pedigree analysis

Pedigree completeness, inbreeding coefficients, and effective numbers of founders, ancestors and founder genomes were calculated using PEDIG software (Boichard, 2002). On the basis of these calculations, population founders are construed to be individuals with no parental data, assuming that this indicates the creation period of that breed. However, because some of the pedigrees are lacking complete data for individuals at time points post-breed-formation, this is not completely applicable in our data set. To avoid confusion in terminology, we will refer to the PEDIG founder calculations instead in terms of ‘earliest documented relative’ (EDR). Thus, we account for individuals that might be founders in the traditional sense, as well as those individuals who have contributed genetically to the breed in more recent years but lack documented pedigree data. Pedigree completeness for the reference population related to each breed was evaluated by first calculating the proportion of known ancestors at each generation and then summing these proportions over all generations, resulting in the number of equivalent complete generations (*g*_e_).

Inbreeding coefficients (*F*) were calculated for each dog in the reference pedigree by computing relationship matrices for the individual and their ancestors (Wiggans et al*.*, 1995), using subsets of all ancestors, ten generations of ancestors, or five generations of ancestors, using the ‘vanrad’ function of PEDIG (Boichard, 2002). The individual *F*-values were averaged across all dogs of a breed that were born within a given year to obtain breed-specific inbreeding coefficients over time. Effective population size (*N*_e_) was calculated as the difference in inbreeding (Δ*F*) between the reference population and the parents of those individuals:
(1)
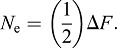


Because the EDR include founder animals as well as recent genetic contributors with missing parentage data, the effective EDR (EDR_e_), defined as the number of equally contributing EDRs expected to produce the amount of genetic diversity observed in the reference population, was calculated as:
(2)
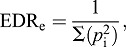


where *p*_i_ is the proportional contribution of the EDR over all descendants in a pedigree (Lacy, 1989). To account for loss of genetic diversity after the foundation of a breed, effective number of ancestors (EDR_a_) was calculated as
(3)
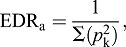


where *p*_k_ is the marginal contribution of each ancestor in the relevant reference pedigree (Boichard et al., 1997). EDR_a_ is the minimum number of ancestors that explain the amount of genetic diversity observed in the reference population. A list of the 100 most influential ancestors and their contributions were also identified for each breed. Because ancestors do not necessarily need to be founder animals, and can be related to one another, the marginal contribution of specific ancestors, i.e. that contribution not yet explained by other ancestors, was considered when evaluating the effect of influential individuals. The ‘prob_orig’ function of PEDIG (Boichard, 2002) was utilized to calculate the EDR_e_, EDR_a_ and ancestor contributions. Additionally, the ratio of EDR_e_/EDR_a_ was calculated because it indicates the occurrence of a bottleneck event when greater than one. Finally, the effective number of EDR genomes (EDR_g_), which measures the probability that an EDR haplotype is still present at a given locus in the reference population and which accounts for all random loss of alleles during segregation and due to genetic drift, was calculated with the ‘segreg’ function of PEDIG (Boichard, 2002) as:
(4)
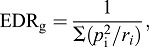


where *r*_i_ is the fraction of an EDR's alleles presumed to have been retained in the population (Lacy, 1989).

### Sample collection and genotyping

Blood samples from purebred dogs were collected from private owners as described previously (Parker et al*.*, 2009) and DNA was isolated using standard phenol-chloroform methods (Sambrook et al*.*, 1989), aliquoted and stored at −80°C. The majority of dogs were registered with the AKC, and AKC registration numbers were used to verify breed affiliation and relatedness. Those that were not registered were pedigree-verified as eligible purebreds. Owners signed an informed consent prior to sample collection in accordance with a National Human Genome Research Institute (NHGRI) Animal Care and Use Committee. Ten dogs from each of 80 breeds, determined to be unrelated within three generations, were genotyped by the Ostrander lab using the Illumina Canine HD SNP chip (Illumina, San Diego, CA). Genotypes were called using Illumina Genome Studio, retaining SNPs with >90% call rate, heterozygous excess of −0.7 to 0.5, and GenTrain score of >0.4.

### SNP-based analysis of population metrics

A homozygous region was calculated as five or more consecutive SNPs, predicted to span at least 70 kb when considering the array average SNP spacing of 14 kb, which were homozygous in an individual dog. These homozygosity parameters would therefore include regions of homozygosity less than the expected minimum length of canine LD of 20 kb (Gray et al*.*, 2009). The total number of such regions is termed regions of homozygosity (RoH). The combined length of all homozygous regions is termed the length of homozygosity (LnH). The RoH and LnH were first calculated for each individual dog, and then combined in sets increasing in size from two to the maximum number of dogs of the same breed. Dogs of the same breed were added one at a time by random selection and RoH and LnH were recalculated as regions of five or more consecutive SNPs that were homozygous in all individuals. In this way, the regions and length of homozygosity common across all individuals of the same breed are termed the shared RoH and shared LnH, respectively. Calculation of shared LnH and shared RoH allowed for a genotype missingness rate of <20% across included dogs with retained complete homozygosity in the remaining genotyped individuals.

The LnH of the first randomly selected dog of each breed was decreased with each additional same-breed dog added to the sequential shared LnH calculations, such that the difference between one-dog private LnH and two-dog shared LnH is exponentially greater than the difference between nine-dog and ten-dog shared LnH. For each breed with ten representatives, exponential rate of decay (*k*) was calculated as:
(5)
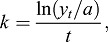


where *t* is the number of additional dogs (i.e. 9), *y*_t_ is the difference in shared LnH between *t*−1 and *t*, and *a* is the amount of LnH lost by the addition of the second dog. Therefore, the number of dogs required to reduce the shared LnH by 1% of the first dog's LnH was calculated with the equation:
(6)
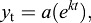


where *y*_t_ is 1% of the first-dog LnH. Likewise, *y*_t_=1 was used to determine the number of dogs required to reduce the shared LnH by only one nucleotide, estimating the point at which the first-dog private LnH will not be decreased further by the addition of more dogs.

The program SNePv1.1 (Barbato et al*.*, 2015) was used to calculate effective population size (*N*_e_) with the SNP genotypes from each breed. Predicted *N*_e_ values for 13 to 995 generations prior to sample age were calculated and values were interpolated using a generation interval of 3.76 years (Windig and Oldenbroek, 2015).

### Whole-genome sequencing data generation

WGSs were compiled using data from 90 purebred dogs representing 80 distinct breeds. Seventy-two breeds were represented by one sequenced dog each, two dogs were sequenced for each of six breeds (Chow Chow, Bernese Mountain Dog, Greyhound, Rottweiler, Scottish Terrier, West Highland White Terrier), and three dogs were sequenced for each of two breeds (Flat-coated Retriever, Irish Water Spaniel). Data were obtained via the Short Read Archive (ncbi.nlm.nih.gov/sra) from previously published studies, or sequenced for this study by the National Institutes of Health (NIH) Intramural Sequencing Center (NISC) using the Illumina TruSeq DNA PCR-Free Protocol (Cat.# FC-121-3001) from DNA samples provided by the Ostrander laboratory (Table S2). Previously unpublished data from 27 sequenced dogs are deposited in the Sequence Read Archive (http://www.ncbi.nlm.nih.gov/sra). Libraries were sequenced on the Illumina HiSeq 2000 platform with 100 bp paired-end fragments of 300-500 bp. Paired data was aligned to the CanFam3.1 reference genome (http://genome.ucsc.edu/cgi-bin/hgGateway?db=canFam3) with the BWA 0.7.10 MEM algorithm (Li and Durbin, 2009), sorted with SAMtools 0.1.10 (Li et al*.*, 2009), and screened for putative PCR duplicate reads with PicardTools 1.119 (https://github.com/broadinstitute/picard).

Sequences were locally realigned based on documented and novel insertions-deletions (Axelsson et al., 2013) using GATK 3.2-2 (DePristo et al*.*, 2011), and training sets of dbSNP and Illumina Canine HD chip positions were used for base quality recalibration. HaplotypeCaller was used in ‘gVCF’ mode (Van der Auwera et al., 2013) to call SNVs for each individual dog, and then jointly across all 90 dogs. GATK best practices and default parameters, together with the initial alignment training sets, were used for variant quality score recalibration of SNVs. Joint-called and compiled variant call files (VCFs) were filtered for CpG islands, gaps and repeats, as annotated in the CanFam3.1 reference dog genome assembly (http://genome.ucsc.edu/cgi-bin/hgGateway?db=canFam3). The resulting set of SNVs was used in subsequent analyses.

### WGS-based analysis of homozygosity

The list of 7,095,427 SNVs from the 90 WGS dogs was pruned for excessive linkage using the ‘indep’ function of PLINK v1.07 (Purcell et al*.*, 2007), with a window size of 50 SNPs, a window step of five SNPs and a variance inflation factor of 2. The pruned variant set of 1,510,327 SNPs was used to calculate RoHs and LnH for each WGS using the ‘homozyg’ function of PLINK (Purcell et al*.*, 2007). To determine optimal conditions for which SNP and single-dog WGS homozygosity measurements are most comparable, homozygosity was calculated per dog from the WGS data with parameters set at a window size of 10 kb, 70 kb, 100 kb and 1000 kb, each with allowed heterozygosity of zero, one, five, or ten heterozygotes per window, equaling 16 total homozygosity conditions for each WGS. The scenario with 70 kb minimum LnH with zero allowed heterozygotes was designed to most closely mimic the parameters set for the SNP chip homozygosity analysis. Shared LnH and shared RoH were calculated for the breeds for which WGS was obtained from two dogs (Chow Chow, Bernese Mountain Dog, Greyhound, Rottweiler, Scottish Terrier, West Highland White Terrier) and three dogs (Flat-coated Retriever, Irish Water Spaniel) utilizing the same methodology and criteria implemented with the SNP data and applied to the pruned WGS.

### Inbreeding coefficients from SNP and WGS data

Inbreeding coefficients were calculated for each of the dogs across 154,230 SNPs from the chip genotyping dataset and 1,510,327 SNPs from the pruned WGS data using the ‘heterozygosity’ function of PLINK v1.07 (Purcell et al*.*, 2007). The within-breed means of the individual dog inbreeding coefficients were used to represent breed-specific inbreeding coefficients for each of the SNP analysis breeds, as well as for the WGS breeds for which more than one dog of a given breed was sequenced (Bernese Mountain Dog, Chow Chow, Flat-coated Retriever, Greyhound, Irish Water Spaniel, Rottweiler, Scottish Terrier, West Highland White Terrier).

### Statistical analysis

The cor.test function of the Hmisc R package was used to calculate Pearson correlation statistics and significance values between SNP-based inbreeding coefficients, RoH and LnH, WGS-based inbreeding coefficients, RoH and LnH, and pedigree-based inbreeding coefficients. Correlation analyses utilized all breeds shared between each pair of data acquisition methods. Because *F*-values were calculated per individual within the WGS and SNP chip analyses, breed values were determined by averaging all contributing dogs of the same breed for each genotyping method. As such, a total of 11 breeds were represented in the pedigree data, 80 breeds in the SNP chip data, and 80 breeds in the WGS data. Fifty breeds were common to the WGS and SNP data, nine breeds to the WGS and pedigree data, and 11 breeds between SNP and pedigree data.
